# Structure of an atypical periplasmic adaptor from a multidrug efflux pump of the spirochete *Borrelia burgdorferi*

**DOI:** 10.1016/j.febslet.2013.06.056

**Published:** 2013-09-17

**Authors:** Nicholas P. Greene, Philip Hinchliffe, Allister Crow, Abdessamad Ababou, Colin Hughes, Vassilis Koronakis

**Affiliations:** Department of Pathology, University of Cambridge, Tennis Court Road, Cambridge CB2 1QP, UK

**Keywords:** MDR, multidrug resistance, IM, inner membrane, OM, outer membrane, MP, membrane proximal, RMSDs, root mean square deviations, HME, heavy metal efflux, MD, molecular dynamics, Antibiotic resistance, Multidrug efflux, Adaptor protein, Crystal structure

## Abstract

Periplasmic adaptor proteins are essential components of bacterial tripartite multidrug efflux pumps. Here we report the 2.35 Å resolution crystal structure of the BesA adaptor from the spirochete *Borrelia burgdorferi* solved using selenomethionine derivatized protein*.* BesA shows the archetypal linear, flexible, multi-domain architecture evident among proteobacteria and retains the lipoyl, β-barrel and membrane-proximal domains that interact with the periplasmic domains of the inner membrane transporter. However, it lacks the α-hairpin domain shown to establish extensive coiled-coil interactions with the periplasmic entrance helices of the outer membrane-anchored TolC exit duct. This has implications for the modelling of assembled tripartite efflux pumps.

## Highlights

• Periplasmic adaptors are essential to tripartite drug efflux pump assembly.
• We present the structure of the periplasmic adaptor BesA from *Borrelia burgdorferi*.
• BesA lacks the α-hairpin shown to underpin exit duct recruitment and pump assembly.
• Recruitment of the TolC exit duct must be different in this pump.
• The BesA structure has implications for proposed models of pump assembly.

## 1 Introduction

Tripartite efflux pumps span the inner and outer membranes of Gram-negative bacteria to expel antibiotics, metals and other noxious molecules [1–3]. They are key to survival and the development of multidrug resistance (MDR). Efflux substrates bind to an inner membrane (IM) transporter, typically a proton antiporter (e.g. *Escherichia coli* AcrB), before delivery to the TolC exit duct [4]. Trimeric TolC [5], anchored in the outer membrane (OM), projects an α-helical barrel into the periplasm where it contacts tip-to-tip [6–8] the 70 Å periplasmic extension of the IM trimeric AcrB transporter [9]. Extensive in vivo site-specific cross-linking and multidomain docking showed that pump assembly is established and stabilised by extensive interactions of both IM and OM components with a ubiquitous third component, the periplasmic adaptor protein [7]. Structural analysis of the *Pseudomonas aeruginosa* adaptor MexA [7,10] and subsequently other multidrug resistance [11,12] and metal [13,14] efflux pump adaptors have revealed conserved flexible, linearly arranged domains: a membrane proximal (MP) domain, a β-barrel domain, a lipoyl domain and an α-helical hairpin. The MP, β-barrel, and lipoyl domains of AcrA contact AcrB while the full length of the 47 Å long α-hairpin establishes extensive coiled-coil interactions with the periplasmic entrance of each TolC protomer [7,15]. The adaptor multidomain structure is conserved throughout proteobacteria, but primary sequence comparison in the spirochete *Borrelia burgdorferi* suggested that a tripartite MDR pump, BesABC, included an adaptor that appeared to lack an α-hairpin [16]. This study reported *besC*, *besA* and *besB* are co-transcribed in an operon, and that insertional disruption of the *besCAB* operon abrogates expression of BesC, BesB and BesA and causes up to 64-fold reduction in the minimal inhibitory concentrations for several antibiotics, SDS and ethidium bromide. Bunikis et al. concluded that BesABC is a functional tripartite RND efflux pump. The absence of an adaptor hairpin in a tripartite pump would be unprecedented and would have implications for understanding the assembly of MDR efflux pumps [2]. We therefore crystallised the BesA adaptor.

## 2 Materials and methods

*Expression of soluble BesA* **–** The BesA sequence indicated a signal sequence and lipidation site (Cys 26) comparable to IM-associated periplasmic adaptors *E. coli* AcrA and *P. aeruginosa* MexA, so *besA* codons 27–318 from *Borrelia burgdorferi* B31 genomic DNA (ATCC 35210) were amplified with forward and reverse primers (5′-GCGCCATATGGTTGGTGATAACAAGCTAGATGAC-3′, 5′-GCGCGGATCCTTAAATATTGCTTTCGGCTGAAAGACCCTC-3′), and the product digested with NdeI/BamHI and cloned into pET28 (Novagen). The resultant pET28-BesA plasmid encodes BesA in which the N-terminal signal sequence is replaced with a hexahistidine tag.

For selenomethionine incorporation, *E. coli* B834 (DE3) was transformed with pET28-BesA, and grown in M9 minimal medium containing 50 μg ml^−1^ selenomethionine. At *A*_600_ 0.6, 0.1 mM IPTG was added and the culture continued for 16 h at 18 °C. Cell pellets were resuspended in 50 mM HEPES pH 8.0, 400 mM NaCl, 5% glycerol, 10 mM MgCl_2_. 1 mM TCEP was included in all buffers. Cells were broken in a cell disruptor (30 000 psi) and centrifuged (150 000×*g*, 1 h at 4 °C). Supernatant was incubated with IMAC resin (Biorad) and 4 mM imidazole (1 h, 4 °C). Resin was washed in buffer A (25 mM HEPES pH 8.0, 500 mM NaCl, 8 mM imidazole) containing 0.1% Triton X-100, then buffer A alone. Protein was eluted in 25 mM HEPES pH 8.0, 200 mM NaCl and 250 mM imidazole and loaded onto a Superdex S75 column equilibrated in 25 mM HEPES pH 8.0, 150 mM NaCl and peak fractions concentrated to 10 mg ml^−1^. To produce native BesA, *E. coli* C41 [17] cells bearing pET28-BesA were grown in 2xTY medium, with expression and purification as above, except the hexahistidine tag was removed with thrombin (Novagen) before loading on a Superdex S75 column. Protein eluted with a molecular weight consistent with monomeric BesA.

Attempts to express correctly folded and localised BesB using N- and C-terminal his- and strep-tags as well as fusions with maltose-binding protein and GFP were repeatedly unsuccessful. A construct, in which the region up to and including the BesB N-terminal transmembrane helix was replaced with the equivalent region of AcrB, also failed to express. Though small amounts of BesC can be expressed, we have not been able to crystallise it.

*Crystallisation and data collection* – BesA crystallisation was conducted using sitting drop vapour diffusion at 15 °C. 2 μl protein solution was mixed with either 1 μl (*C*2 and *P*2_1_ crystal forms) or 2 μl (*P*2_1_2_1_2_1_ crystal form) crystallisation reagent and equilibrated against 500 μl reagent. The *P*2_1_2_1_2_1_ form was crystallised using 100 mM phosphate/citrate pH 4.2, 100 mM LiSO_4_, 16% PEG 1000. *C*2 and *P*2_1_ forms were obtained using thrombin-treated protein with crystallization reagents comprising 100 mM phosphate/citrate pH 4.2, 30% PEG 300, and 100 mM citrate pH 4.7, 9% PEG 6000, respectively. Crystals grew to a maximum size of 0.3 mm × 0.2 mm × 0.2 mm in four days.

Crystals were cryoprotected by stepwise addition of 12 μl cryoprotectant to the crystallisation drop, before being flash-frozen in liquid nitrogen. Cryoprotectant for *P*2_1_2_1_2_1_ crystals comprised 100 mM phosphate/citrate pH 4.2, 200 mM LiSO_4_, 15% PEG 1000 and 25% PEG 400. Cryoprotectants for the native *C*2 and *P*2_1_ crystal forms contained 100 mM phosphate/citrate pH 4.2, 15% PEG 300, 25% glycerol and 100 mM citrate pH 4.2, 12% PEG 6000, 25% glycerol, respectively.

X-ray diffraction data were collected at 100 K on beamline ID29 at the European Synchrotron Radiation Facility (Grenoble, France), after screening on beamline I02 at Diamond Light Source (Oxford, UK).

*Crystal structure determination and analysis* – X-ray data sets were indexed and integrated using iMosflm [18] and scaled using Scala or Aimless in the CCP4 suite [19]. Crystallographic phases for the 2.6 Å selenomethionine dataset were obtained by Single-wavelength anomalous diffraction (SAD). Selenium sites were identified using HYSS [20], and phases calculated with Phaser [21]. Density modification was performed in Parrot [22] using 4-fold non-crystallographic symmetry (NCS) and an initial model of BesA built with Buccaneer [23] with four monomers in the asymmetric unit. The structure was completed with manual model-building in Coot [24] and refinement in Phenix [25] using NCS restraints between each of the three BesA domains. Upon completion of the selenoprotein model, refinement was switched to the isomorphous high-resolution data, maintaining the same ‘free’ reflection list. Model building and refinement employed Coot [24] and Refmac [26], with NCS restraints as before.

Structures of *C*2 and *P*2_1_ crystal forms were determined by molecular replacement using Molrep [27] or Phaser [21] with a BesA monomer as a probe. Refinement was performed as above except the *C*2 data initially benefitted from jelly body refinement and did not make use of NCS restraints. Validation was assisted by Molprobity [28] and Procheck [29]. The final model of the high resolution *C*2 crystal form is the only dataset for which a reasonable atomic model could be built for the residual loop (i.e. residues 106–114). The structures have been deposited in the PDB with accession codes 4KKS, 4KKT and 4KKU.

Root mean square deviations (RMSDs) were calculated using PDBeFold (http://www.ebi.ac.uk/msd-srv/ssm) [30]. Homology models of BesC and BesB were created using Phyre [31], based on TolC (1EK9) and AcrB (1T9Y), respectively. Figures were prepared using PyMol [32].

*Molecular dynamics (MD) simulations* – Energy minimizations and MD simulations were performed using Amber11 [33] as previously [34]. The protein formal charge of +6e was neutralized by adding Cl^−^ ions. The simulated system consists of 276 residues, 6 Cl^−^ ions, and 18,589 water molecules, making a total of 60 159 atoms. MD trajectory calculations of 23 ns were performed on the Darwin cluster (http://www.hpc.cam.ac.uk/services/darwin.html).

## 3 Results and discussion

**Fig. 1:**
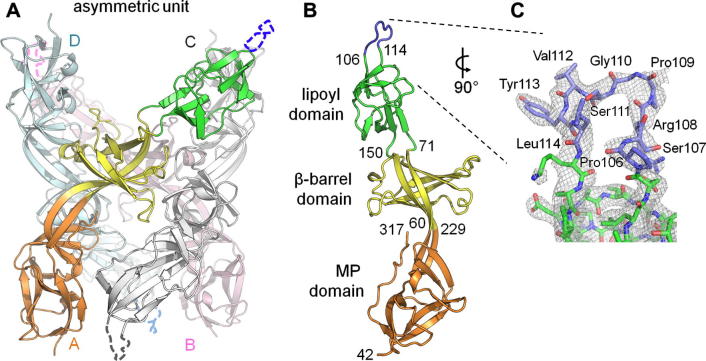
Architecture of the *Borrelia* periplasmic adaptor BesA. (A) Arrangement of 4 monomers in the *P*2_1_2_1_2_1_ crystal lattice. Chain A is coloured by domain in orange (membrane proximal), yellow (β-barrel) and green (lipoyl). Dotted lines indicate the likely position of (unmodelled) residues 106–114 which have poorly defined electron density. (B) BesA monomer from the *C*2 space group. (C) The loop at the lipoyl domain tip represented as sticks with surrounding electron density (grey) contoured at 1σ. For this presentation the monomer is rotated 90° to (B).

**Fig. 2:**
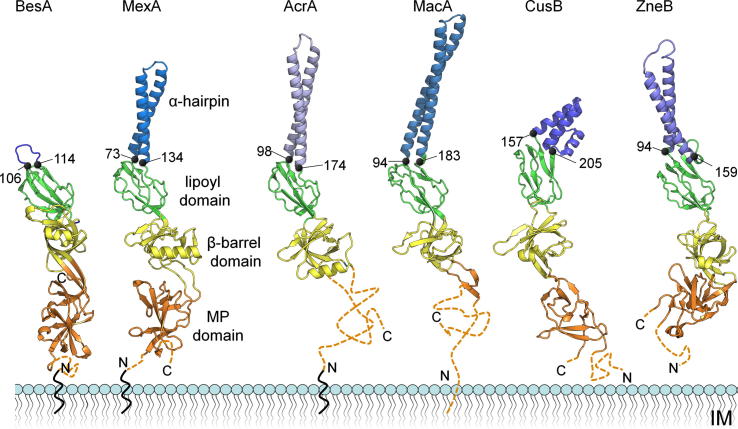
Structural comparison of periplasmic adaptor proteins. BesA structure compared to known drug efflux pump adaptors MexA (*P. aeruginosa*) and AcrA (*E. coli*), MacA (*E. coli* macrolide efflux pump) and the metal efflux pump adaptors CusB (*E. coli*) and ZneB (*C. metallidurans*). MP domains of BesA, MexA and AcrA have an N-terminal lipoyl attachment site anchoring the adaptor in the IM. Dotted orange lines indicate unobserved MP domain terminal regions.

The crystal structure of the complete BesA protein was solved by SAD in three space groups, *P*2_1_2_1_2_1_ (2.35 Å resolution), *P*2_1_ (2.5 Å) and *C*2 (2.6 Å) (Table 1), in which either a monomer (*C*2) or four intertwined head-to-tail monomers (*P*2_1_ and *P*2_1_2_1_2_1_, Fig. 1A) are present in the asymmetric unit. Given the protein is membrane localised by an N-terminal lipidation, this head-to-tail arrangement, also seen in the isolated CusB structure [14], cannot be physiologically relevant. The BesA monomer shows the archetypal, multidomain adaptor topology, with the MP, β-barrel, and lipoyl domains in linear arrangement (coloured orange, yellow and green, respectively, in Fig. 1A and B). However, the expected final α-hairpin domain is completely absent, confirming what was predicted from sequence analysis [16], leaving only a nine residue loop (Pro106 to Leu114, blue in Fig. 1B) linking the two halves of the lipoyl domain. Density for this loop is visible only in the *C*2 monomer (Fig. 1C) where it is stabilised by the loop of a neighbouring monomer in the crystal lattice, but high B-factors here and poorly defined density for the *P*2_1_2_1_2_1_ or *P*2_1_ monomers indicate the small loop is highly flexible. The BesA architecture is distinct, the other adaptor structures have a well defined α-helical hairpin up to 67 Å/90 residues long (Fig. 2) [10–14]. The *B. burgdorferi* genome reveals no other adaptors that could perform the function of BesA, and no hairpin-encoding gene in the vicinity of the *besCAB* operon. However we cannot exclude that a novel, unidentified factor exists that could compensate for the lack of an α-hairpin in BesA.

**Fig. 3:**
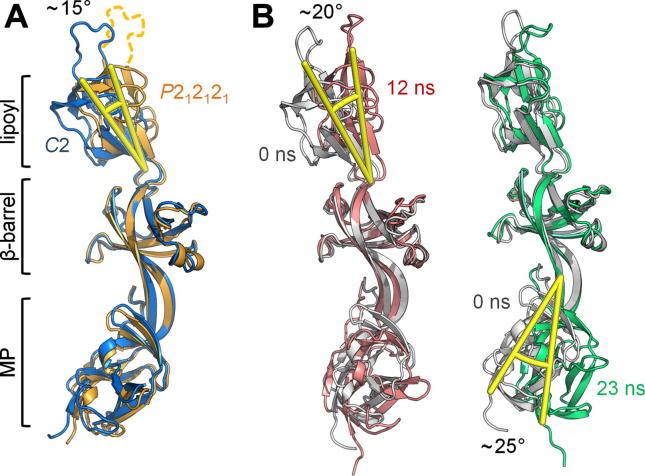
Interdomain movement of BesA hinged at the flexible linkers. Structures were superposed over residues 60–71 and 150–229 (i.e. the β-barrel domain) using superpose in the CCP4 suite. (A) Comparison of BesA monomers crystallised in *C*2 (blue) and *P*2_1_2_1_2_1_ (orange) space groups. Relative interdomain movement is measured in degrees. Unmodelled loop residues are shown as dotted lines. (B) Molecular dynamics (MD) simulation of BesA. The starting model (0 ns, grey from *P*2_1_2_1_2_1_) is superposed to the main chain of snapshots from the MD trajectories at 12 ns (red) and 23 ns (green).

The conserved MP, β-barrel, and lipoyl domains of BesA have low RMSDs compared to counterparts in other MDR [7,10–12] and heavy metal efflux (HME) pump adaptors [13,14] (Supplemental Table 1). This conforms to the view they play a key role in pump assembly and function, particularly in binding to the large periplasmic extension of the IM transporters. As would be expected in this case, primary sequence analysis indicates the cognate interacting transporter domains of BesB and other transporters are likewise structurally conserved (Supplemental Table 2) [16]. In both the data-based assembly model of AcrAB-TolC [7] and the in vitro co-crystal structure of an adaptor-transporter (CusBA) subcomplex [35] tripartite pump assembly is facilitated by the flexibility of the adaptor’s serially-linked domains [11,13], which was confirmed by MD analysis of the closely related MexA [36]. This ‘hinge’ flexibility at the inter-domain linkers is also evident in the BesA variant space groups which show movement of the lipoyl domain, relative to the other domains (Fig. 3A). We further analysed BesA interdomain dynamics by performing a 23 ns MD simulation (Fig. 3B) of a single monomer in aqueous solution. Two major motions are observed ‘hinged’ at the interdomain linkers, i.e. independent movement of both the lipoyl domain (Fig. 3B, *left*) and the MP domain (Fig. 3B, *right*) relative to the β-barrel domain.

**Fig. 4:**
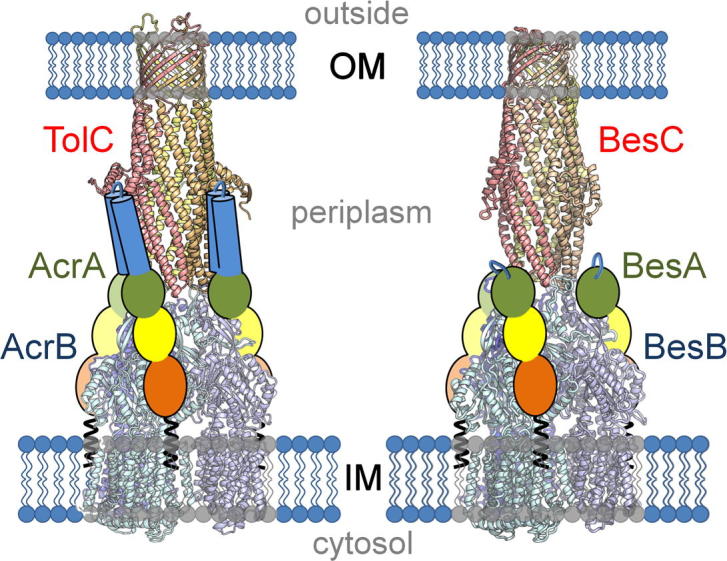
Predicted assembly of the tripartite BesABC pump of *B. burgdorferi*. *Left*, assembled *E. coli* TolC (red)-AcrA (coloured as in Fig. 2)-AcrB (blue) pump, based on in vivo site-specific cross-linking and data-based multidomain docking [7]. *Right***,** predicted assembly of *B. burgdorferi* BesC (homology model, red)-BesA (coloured as in Fig. 2)-BesB (homology model, blue), based on the *E. coli* model. The simplest 1:1:1 ratio of BesA-BesB-BesC is shown, based on AcrA-AcrB-TolC [7]. A 2:1:1 ratio has also been suggested in which 6 adaptors form a ring round the transporter mediated by contacts through the β-barrel and lipoyl domains, as seen in the in vitro co-crystallised CusBA subcomplex [3,35,37]. The absence of an adaptor α-hairpin in *Borrelia* BesA is not compensated by extra domains in the cognate IM (BesB) or OM (BesC) pump components.

This conserved organisation, domain structure and inter-domain flexibility indicate a common adaptor function in proteobacteria and spirochetes. The absence of an α-hairpin in the BesA adaptor therefore has significant implications for tripartite efflux pump assembly. Our data-based model of the *E. coli* AcrAB-TolC pump suggests the IM transporter and OM channel contact each other via their juxtaposed apical α-helices, mediated by extensive interactions of these two components with the four adaptor domains (Fig. 4, *left*) [7]. Based on this model our predicted assembly of the *Borrelia* pump indicates interaction between the exit duct and transporter would be essential to provide a continuous tunnel for substrate to bypass the periplasm and exit the cell (Fig. 4, *right*). In this consistent view of assembly the adaptor lipoyl domain would be well positioned to contact the exit duct (Fig. 4), as it would also be in the predicted HME CusBAC assembly [35,37] in which the adaptor sits further up the IM transporter than is calculated for drug efflux adaptors AcrA/MexA. This suggests a key role for the lipoyl domain in the spirochete BesABC pump not only in interacting with the transporter TolC-docking domains, as in the *E. coli/Pseudomonas* pumps [7], but also in stabilising TolC interaction. Otherwise, there would be very little contact between adaptor and OM exit duct (Fig. 4, *right*). The BesA architecture is incompatible with any model in which adaptor hairpins are suggested to form homotypic interactions to create a tunnel that contacts TolC tip-to-tip (Supplemental Fig. 1, *left*) [38], as in such a model the predicted assembly is reliant on the hairpin to form a sealed channel between separated IM and OM components (Supplemental Fig. 1, *right*). Indeed, this model (Supplemental Fig. 1, *left*) is inconsistent with in vivo cross-linking data showing AcrB and TolC interact [6,8], in vivo cross-linking that shows the full length of the AcrA hairpin interacts with the periplasmic entrance of TolC [7,15] and the CusBA crystal structure in which interactions between CusB adaptors are mediated not through the α-hairpin domains but through the β-barrel and lipoyl domains [3,35,37].

The high resolution structure of BesA establishes unequivocally an important divergence from the paradigm adaptor structure. Primary sequences of other putative spirochete adaptors suggest the BesA architecture may not be unique, as adaptor sequences lacking the α-hairpin coding region can be identified in *Borrelia*, *Treponema* and *Spirochaeta* (Supplemental Fig. 2). Indeed, comparable primary sequences are also evident rarely in proteobacteria (e.g. *Legionella*). This indicates that BesABC and most likely other similar pumps are assembled without the extensive coiled-coil interactions shown to be central to the otherwise closely related tripartite efflux pumps of *E. coli* and *P. aeruginosa*. It is therefore possible that despite close structural similarity among components different pumps require distinct component interactions.

## Figures and Tables

**Table 1:** Crystallographic data and refinement statistics.

	BesA – SeMet	BesA – SeMet	BesA – native	BesA – native
*Data collection*				
Space group	*P*2_1_2_1_2_1_	*P*2_1_2_1_2_1_	*P*2_1_	*C*2
Cell dimensions				
*a*, *b*, *c* (Å)	73.71, 151.93, 155.46	73.74, 152.13, 155.49	78.20, 73.74, 152.23	83.20, 52.50, 76.53
α, β, γ (°)	90, 90, 90	90, 90, 90	90, 98.06, 90	90.0, 98.04, 90.0
Wavelength (Å)	0.9787	0.9787	0.9800	0.9800
Resolution (Å)	66.6–2.60	77.87–2.35	73.74–2.53	44.3–2.60
	(2.68–2.60)	(2.40–2.35)	(2.60–2.53)	(2.72–2.60)
*R*_sym_	0.116 (0.646)	0.106 (0.715)	0.0755 (0.171)	0.036 (0.147)
*I*/σ(*I)*	12.8 (3.6)	12.3 (2.2)	10.0 (5.1)	16.2 (4.4)
Completeness (%)	99.91 (99.64)	98.53 (89.55)	95.6 (91.3)	95.25 (78.35)
Redundancy	6.0 (5.1)	10.7 (6.0)	3.6 (3.5)	3.3 (2.7)
*Refinement*				
Resolution (Å)		77.87–2.35	73.28–2.53	44.3–2.6
No. reflections		68,894	52,051	9,270
*R*_work_/*R*_free_		0.236/0.264	0.257/0.286	0.206/0.286
No. atoms				
Protein		8,284	8156	2134
Ligand/ion		0	0	5
Water		178	111	18
*B*-factors (Å^2^)				
Protein		42.70	36.9	76.1
Ligand/ion		–	–	96.5
Water		34.60	23.2	59.8
R.m.s. deviations				
Bond lengths (Å)		0.018	0.015	0.011
Bond angles (°)		1.81	1.66	1.52
Ramachandran plot				
Most favoured (%)		96.47	98.17	96.70
Outliers (%)		0.00	0.00	1.10

Values in parenthesis are for the highest resolution shell.

## Appendix A Supplementary data

Supplementary data 1 This document file contains supplementary figures and tables.

## References

[1] Koronakis V., Eswaran J., Hughes C. (2004). Structure and function of TolC: the bacterial exit duct for proteins and drugs. Annu. Rev. Biochem..

[2] Eswaran J., Koronakis E., Higgins M.K., Hughes C., Koronakis V. (2004). Three’s company: component structures bring a closer view of tripartite drug efflux pumps. Curr. Opin. Struct. Biol..

[3] Hinchliffe, P., Symmons, M.F., Hughes, C. and Koronakis, V. (2013) Structure and operation of bacterial tripartite pumps. Annu. Rev. Microbiol. 67, http://dx.doi.org/10.1146/annurev-micro-092412-155718. 10.1146/annurev-micro-092412-155718 23808339

[4] Pos K.M. (2009). Drug transport mechanism of the AcrB efflux pump. Biochim. Biophys. Acta.

[5] Koronakis V., Sharff A., Koronakis E., Luisi B., Hughes C. (2000). Crystal structure of the bacterial membrane protein TolC central to multidrug efflux and protein export. Nature.

[6] Touze T., Eswaran J., Bokma E., Koronakis E., Hughes C., Koronakis V. (2004). Interactions underlying assembly of the *Escherichia coli* AcrAB-TolC multidrug efflux system. Mol. Microbiol..

[7] Symmons M.F., Bokma E., Koronakis E., Hughes C., Koronakis V. (2009). The assembled structure of a complete tripartite bacterial multidrug efflux pump. Proc. Natl. Acad. Sci. USA.

[8] Tamura N., Murakami S., Oyama Y., Ishiguro M., Yamaguchi A. (2005). Direct interaction of multidrug efflux transporter AcrB and outer membrane channel TolC detected via site-directed disulfide cross-linking. Biochemistry.

[9] Murakami S., Nakashima R., Yamashita E., Yamaguchi A. (2002). Crystal structure of bacterial multidrug efflux transporter AcrB. Nature.

[10] Higgins M.K., Bokma E., Koronakis E., Hughes C., Koronakis V. (2004). Structure of the periplasmic component of a bacterial drug efflux pump. Proc. Natl. Acad. Sci. USA.

[11] Mikolosko J., Bobyk K., Zgurskaya H.I., Ghosh P. (2006). Conformational flexibility in the multidrug efflux system protein AcrA. Structure.

[12] Yum S., Xu Y., Piao S., Sim S.H., Kim H.M., Jo W.S., Kim K.J., Kweon H.S., Jeong M.H., Jeon H., Lee K., Ha N.C. (2009). Crystal structure of the periplasmic component of a tripartite macrolide-specific efflux pump. J. Mol. Biol..

[13] De Angelis F., Lee J.K., O’Connell J.D., Miercke L.J., Verschueren K.H., Srinivasan V., Bauvois C., Govaerts C., Robbins R.A., Ruysschaert J.M., Stroud R.M., Vandenbussche G. (2010). Metal-induced conformational changes in ZneB suggest an active role of membrane fusion proteins in efflux resistance systems. Proc. Natl. Acad. Sci. USA.

[14] Su C.C., Yang F., Long F., Reyon D., Routh M.D., Kuo D.W., Mokhtari A.K., Van Ornam J.D., Rabe K.L., Hoy J.A., Lee Y.J., Rajashankar K.R., Yu E.W. (2009). Crystal structure of the membrane fusion protein CusB from *Escherichia coli*. J. Mol. Biol..

[15] Lobedanz S., Bokma E., Symmons M.F., Koronakis E., Hughes C., Koronakis V. (2007). A periplasmic coiled-coil interface underlying TolC recruitment and the assembly of bacterial drug efflux pumps. Proc. Natl. Acad. Sci. USA.

[16] Bunikis I., Denker K., Ostberg Y., Andersen C., Benz R., Bergstrom S. (2008). An RND-type efflux system in *Borrelia burgdorferi* is involved in virulence and resistance to antimicrobial compounds. PLoS Pathog..

[17] Miroux B., Walker J.E. (1996). Over-production of proteins in *Escherichia coli*: mutant hosts that allow synthesis of some membrane proteins and globular proteins at high levels. J. Mol. Biol..

[18] Battye T.G., Kontogiannis L., Johnson O., Powell H.R., Leslie A.G. (2011). IMOSFLM: a new graphical interface for diffraction-image processing with MOSFLM. Acta Crystallogr. D Biol. Crystallogr..

[19] Winn M.D., Ballard C.C., Cowtan K.D., Dodson E.J., Emsley P., Evans P.R., Keegan R.M., Krissinel E.B., Leslie A.G., McCoy A., McNicholas S.J., Murshudov G.N., Pannu N.S., Potterton E.A., Powell H.R., Read R.J., Vagin A., Wilson K.S. (2011). Overview of the CCP4 suite and current developments. Acta Crystallogr. D Biol. Crystallogr..

[20] Grosse-Kunstleve R.W., Adams P.D. (2003). Substructure search procedures for macromolecular structures. Acta Crystallogr. D Biol. Crystallogr..

[21] McCoy A.J., Grosse-Kunstleve R.W., Adams P.D., Winn M.D., Storoni L.C., Read R.J. (2007). Phaser crystallographic software. J. Appl. Crystallogr..

[22] Zhang K.Y., Cowtan K., Main P. (1997). Combining constraints for electron-density modification. Methods Enzymol..

[23] Cowtan K. (2006). The Buccaneer software for automated model building 1. Tracing protein chains. Acta Crystallogr. D Biol. Crystallogr..

[24] Emsley P., Cowtan K. (2004). Coot: model-building tools for molecular graphics. Acta Crystallogr. D Biol. Crystallogr..

[25] Adams P.D., Afonine P.V., Bunkoczi G., Chen V.B., Davis I.W., Echols N., Headd J.J., Hung L.W., Kapral G.J., Grosse-Kunstleve R.W., McCoy A.J., Moriarty N.W., Oeffner R., Read R.J., Richardson D.C., Richardson J.S., Terwilliger T.C., Zwart P.H. (2010). PHENIX: a comprehensive Python-based system for macromolecular structure solution. Acta Crystallogr. D Biol. Crystallogr..

[26] Murshudov G.N., Vagin A.A., Dodson E.J. (1997). Refinement of macromolecular structures by the maximum-likelihood method. Acta Crystallogr. D Biol. Crystallogr..

[27] Vagin A., Teplyakov A. (1997). MOLREP: an Automated Program for Molecular Replacement. J. Appl. Crystallogr..

[28] Chen V.B., Arendall W.B., Headd J.J., Keedy D.A., Immormino R.M., Kapral G.J., Murray L.W., Richardson J.S., Richardson D.C. (2010). MolProbity: all-atom structure validation for macromolecular crystallography. Acta Crystallogr. D Biol. Crystallogr..

[29] Laskowski R.A., MacArthur M.W., Moss D.S., Thornton J.M. (1993). PROCHECK: a program to check the stereochemical quality of protein structures. J. Appl. Crystallogr..

[30] Krissinel E., Henrick K. (2004). Secondary-structure matching (PDBeFold), a new tool for fast protein structure alignment in three dimensions. Acta Crystallogr. D Biol. Crystallogr..

[31] Kelley L.A., Sternberg M.J. (2009). Protein structure prediction on the Web: a case study using the Phyre server. Nat. Protoc..

[32] DeLano W.L. (2002). The PyMOL user’s manual.

[33] Case D.A., Darden T.A., Cheatham T.E., Simmerling C.L., Wang J., Duke R.E., Luo R., Walker R.C., Zhang W., Merz K.M., Roberts B., Wang B., Hayik S., Roitberg A., Seabra G., Kolossváry I., Wong K.F., Paesani F., Vanicek J., Liu J., Wu X., Brozell S.R., Steinbrecher T., Gohlke H., Cai Q., Ye X., Wang J., Hsieh M.-J., Cui G., Roe D.R., Mathews D.H., Seetin M.G., Sagui C., Babin V., Luchko T., Gusarov S., Kovalenko A., Kollman P.A. (2010). AMBER 11.

[34] Ritco-Vonsovici M., Ababou A., Horton M. (2007). Molecular plasticity of beta-catenin: new insights from single-molecule measurements and MD simulation. Protein Sci..

[35] Su C.C., Long F., Zimmermann M.T., Rajashankar K.R., Jernigan R.L., Yu E.W. (2011). Crystal structure of the CusBA heavy-metal efflux complex of *Escherichia coli*. Nature.

[36] Vaccaro L., Koronakis V., Sansom M.S. (2006). Flexibility in a drug transport accessory protein: molecular dynamics simulations of MexA. Biophys. J..

[37] Long F., Su C.-C., Lei H.-T., Bolla J.R., Do S.V., Edward W.Y. (2012). Structure and mechanism of the tripartite CusCBA heavy-metal efflux complex. Phil. Trans. Royal Soc. B: Biol. Sci..

[38] Xu Y., Lee M., Moeller A., Song S., Yoon B.Y., Kim H.M., Jun S.Y., Lee K., Ha N.C. (2011). Funnel-like hexameric assembly of the periplasmic adapter protein in the tripartite multidrug efflux pump in Gram-negative bacteria. J. Biol. Chem..

