# Supplementary material for: Structure of an atypical periplasmic adaptor from a multidrug efflux pump of the spirochete Borrelia burgdorferi
Source: FEBS Lett. 2013 Sep 17;587(18):2984–8. doi: 10.1016/j.febslet.2013.06.056 (PMC3807786; doi:10.1016/j.febslet.2013.06.056)
Supplement: Supplementary data 1 — This document file contains supplementary figures and tables. [file mmc1.docx]

Supplemental material for

Structure of an atypical periplasmic adaptor from a multidrug efflux pump of the spirochete *Borrelia burgdorferi*

**Nicholas P. Greene^1^, Philip Hinchliffe^1^, Allister Crow, Abdessamad Ababou,
 Colin Hughes, Vassilis Koronakis^2^**

Department of Pathology, University of Cambridge, Tennis Court Road, Cambridge, CB2 1QP, UK

^1^These authors contributed equally to this work

^2^Corresponding author: Vassilis Koronakis, Tel: +44 (0)1223 339766; E-mail: [vk103@cam.ac.uk](mailto:vk103@cam.ac.uk)

**This PDF includes:**

**Supplemental Data**

**S1. Supplemental tables
S2. Supplemental figures**

**Supplemental references**

**S1. Supplemental tables**

| **Supplemental Table 1. RMSDs^1^ between BesA subdomains and the equivalent domains of structurally characterised adaptor homologues.** | | | | | |
| --- | --- | --- | --- | --- | --- |
|  | **adaptor** | | | | |
| **BesA** | **CusB** | **MexA** | **AcrA** | **ZneB** | **MacA** |
| **lipoyl** (67)^2^ | 1.84 (69) | 1.21 (68) | 1.21 (69) | 1.51 (82) | 0.99 (74) |
| **β-barrel** (87) | 2.04 (93) | 2.21 (106) | 1.74 (81) | 1.85 (91) | 1.74 (90) |
| **MP** (102) | 1.79 (80) | 4.22 (85) | – (–)^3^ | 2.82 (96) | – (–)^4^ |
| ^1^RMSDs calculated over Cα atoms using the PDBeFold server. ^2^number of residues for each domain resolved in the corresponding crystal structure is indicated in parenthesis ^3^AcrA MP domain was removed prior to crystallisation ^4^MacA does not have observable density for the MP domain | | | | | |

| **Supplemental Table 2. Antiporter alignment scores** | | | | |
| --- | --- | --- | --- | --- |
|  | **CusA** | **AcrB** | **MexB** |  |
| **BesB** | 94^1^ / 45^2^ | 93^1^ / 45^2^ | 90^1^ / 47^2^ |  |
| **CusA** | – | 92^1^ / 42^2^ / 3.16^3^ | 93^1^ / 42^2^ / 2.93^3^ |  |
| **AcrB** | – | – | 99^1^ / 83^2^ / 1.99^3^ |  |

^1^Sequence alignment scores from the T-Coffee server ([1](#_ENREF_1)).
^2^positive matches identified by the BLAST server (<http://blast.ncbi.nlm.nih.gov/>) ([2](#_ENREF_2)).

^3^Structural RMSDs calculated over Cα atoms using the PDBeFold server.

**S2. Supplemental Figures**


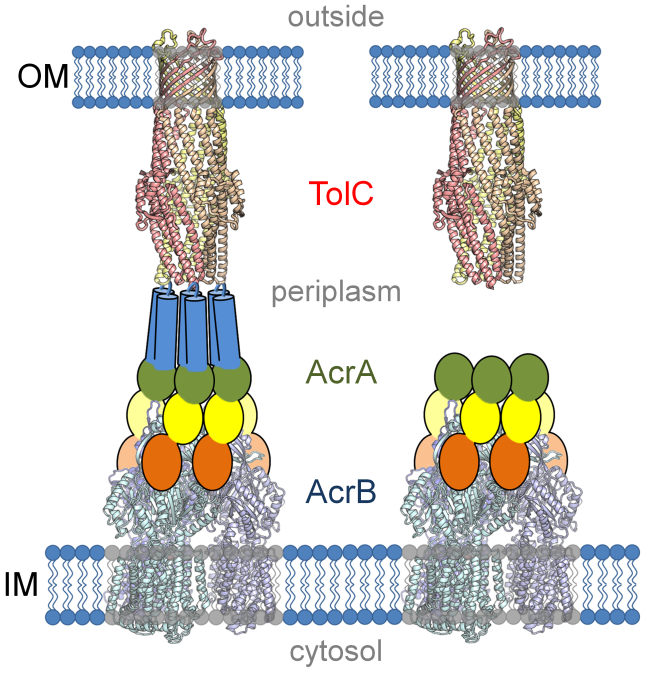


**Supplemental Figure 1. Assembly of the AcrA-AcrB-TolC efflux pump as proposed by Xu *et al* (2011) (**[**3**](#_ENREF_3)**).***Left*, assembly of AcrA-AcrB-TolC based on a tip-to-tip interaction between AcrA hairpins and TolC as proposed by Xu *et al* (2011) ([3](#_ENREF_3)). *Right*, the hairpins of AcrA have been removed from the model (*left*) to highlight the separation between TolC and AcrB that is intrinsic to the non data-driven model from this proposal. As a consequence, in contrast to the data-driven multdomain docking presented in Figure 3, this appears untenable in light of the BesA structure which mimics the right hand situation. The Xu *et al* model is derived from non-native fusion proteins and the hexameric crystal structure of MacA in isolation. It is not compatible with data demonstrating AcrB and TolC interact in vivo ([4](#_ENREF_4),[5](#_ENREF_5)), nor the structure of an adaptor assembled with its cognate transporter (CusBA), in which interactions are not mediated through homotypic α-hairpin contacts but through interactions between the β-barrel and lipoyl domains ([6](#_ENREF_6),[7](#_ENREF_7)).

**
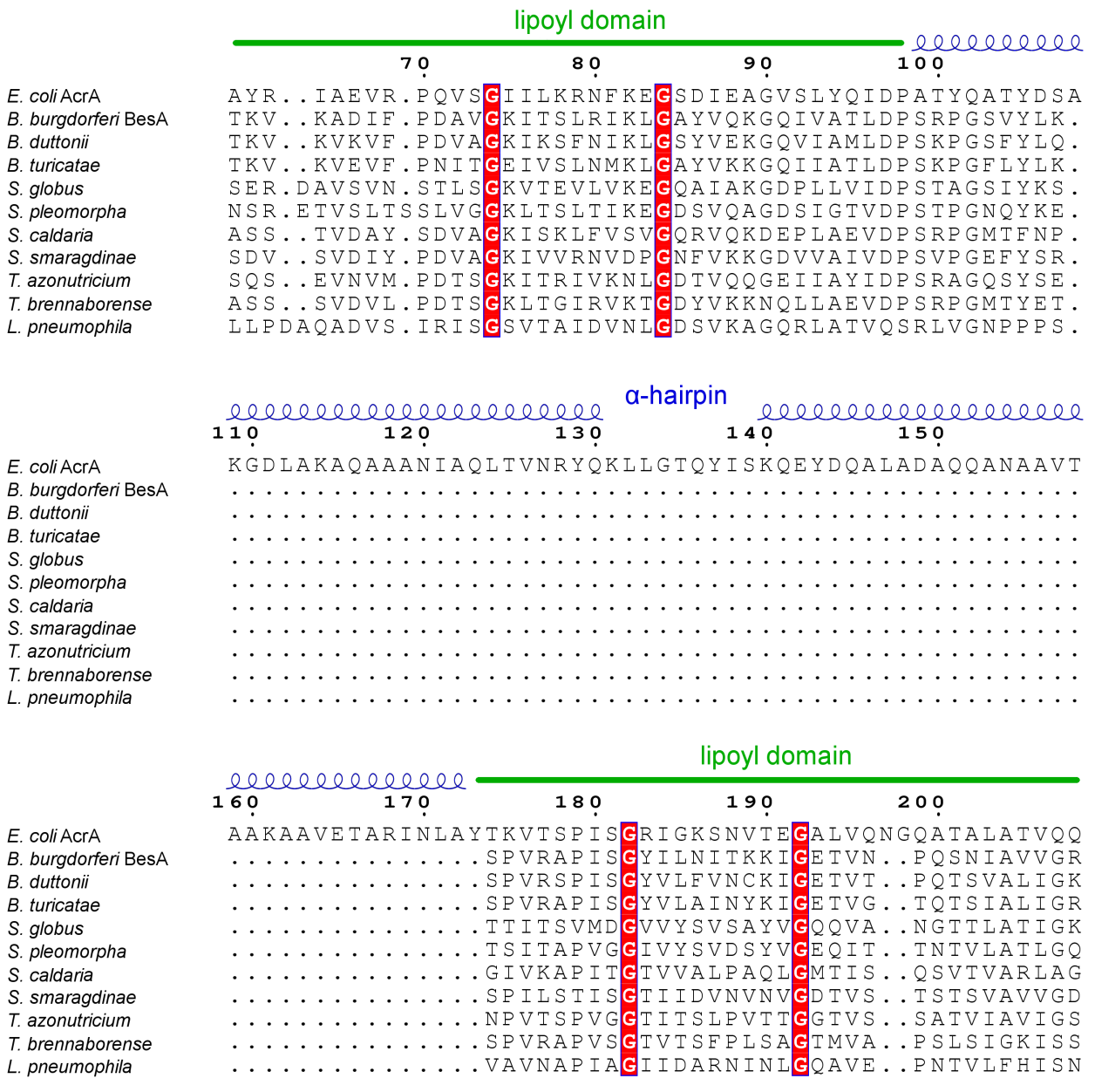
**

**Supplemental Figure 2. Sequence alignment of *E. coli* AcrA with a representative sample of adaptors lacking an α-hairpin.** Alignment was performed over the whole sequence, but for clarity only the region corresponding to the lipoyl and α-hairpin domains of AcrA (indicated above alignment) are shown. Numbering corresponds to full-length AcrA. Strictly conserved residues are in red. The sequences are from *E.coli* (AcrA) and the spirochetes *Borrelia burgdorferi* (BesA), *Borrelia* *duttoni* (uniprot accession number B5RLL5), *Borrelia* *turicatae* (A1QYU0), *Sphaerochaeta* *globus* (F0RYL2), *Sphaerochaeta* *pleomorpha* (G8QQV8), *Spirochaeta* *caldari* (F8EWX3), *Spirochaeta* *smaragdinae* (E1RCF3), *Treponema* *azonutricium* (F5YAE8) and *Treponema brennaborense* (F4LLX5). Also presented is an adaptor from the proteobacterium *Legionella pneumophila* (Q5X3G2).

**Supplemental References**

1. Notredame, C., Higgins, D. G., and Heringa, J. (2000) T-Coffee: A novel method for fast and accurate multiple sequence alignment. *J. Mol. Biol.* **302**, 205-217

2. Altschul, S. F., Gish, W., Miller, W., Myers, E. W., and Lipman, D. J. (1990) Basic local alignment search tool. *J. Mol. Biol.* **215**, 403-410

3. Xu, Y., Lee, M., Moeller, A., Song, S., Yoon, B. Y., Kim, H. M., Jun, S. Y., Lee, K., and Ha, N. C. (2011) Funnel-like hexameric assembly of the periplasmic adapter protein in the tripartite multidrug efflux pump in gram-negative bacteria. *J. Biol. Chem.* **286**, 17910-17920

4. Tamura, N., Murakami, S., Oyama, Y., Ishiguro, M., and Yamaguchi, A. (2005) Direct interaction of multidrug efflux transporter AcrB and outer membrane channel TolC detected via site-directed disulfide cross-linking. *Biochemistry* **44**, 11115-11121

5. Touze, T., Eswaran, J., Bokma, E., Koronakis, E., Hughes, C., and Koronakis, V. (2004) Interactions underlying assembly of the Escherichia coli AcrAB-TolC multidrug efflux system. *Mol. Microbiol.* **53**, 697-706

6. Long, F., Su, C.-C., Lei, H.-T., Bolla, J. R., Do, S. V., and Edward, W. Y. (2012) Structure and mechanism of the tripartite CusCBA heavy-metal efflux complex. *Philosophical Transactions of the Royal Society B: Biological Sciences* **367**, 1047-1058

7. Su, C. C., Long, F., Zimmermann, M. T., Rajashankar, K. R., Jernigan, R. L., and Yu, E. W. (2011) Crystal structure of the CusBA heavy-metal efflux complex of Escherichia coli. *Nature* **470**, 558-562
